# Serum microRNAs in Systemic Sclerosis, Associations with Digital Vasculopathy and Lung Involvement

**DOI:** 10.3390/ijms231810731

**Published:** 2022-09-14

**Authors:** Anna Wajda, Marcela Walczyk, Ewa Dudek, Barbara Stypińska, Aleksandra Lewandowska, Katarzyna Romanowska-Próchnicka, Marek Chojnowski, Marzena Olesińska, Agnieszka Paradowska-Gorycka

**Affiliations:** 1 Department of Molecular Biology, National Institute of Geriatrics, Rheumatology and Rehabilitation, 02-637 Warsaw, Poland; 2 Department of Connective Tissue Diseases, National Institute of Geriatrics, Rheumatology and Rehabilitation, 02-637 Warsaw, Poland; 3 Department of General and Experimental Pathology with Centre for Preclinical Research and Technology (CEPT), Medical University of Warsaw, 02-097 Warsaw, Poland

**Keywords:** systemic sclerosis, microRNA

## Abstract

**Background and aims:** Systemic sclerosis (SSc) is an autoimmune, rare multisystem chronic disease that is still not well-understood aetiologically and is challenging diagnostically. In the literature, there are ever-increasing assumptions regarding the epigenetic mechanisms involved in SSc development; one of them is circulating microRNAs. Many of them regulate TLR pathways and are significant in autoimmune balance. The aim of this study was to determine profile expression of selected microRNAs in SSc patients, including miR-126, -132, -143, -145, -155, -181a, -29a and -3148, in comparison to healthy controls. **Methods:** Serum microRNAs were isolated from 45 patients with SSc and 57 healthy donors (HC). Additionally, SSc patients were considered in the aspect of disease subtype, including diffuse systemic sclerosis (dcSSc) and limited systemic sclerosis (lcSSc). **Results:** miR-3148 was detected neither in the serum of HC nor in SSc patients. All of the rest of the analyzed microRNAs, excluding miR-126, miR-29a and miR-181a, were significantly upregulated in SSc patients in comparison to HC. However, miR-181a has been revealed only in the serum of patients with lcSSc but not dcSSc. Moderate positive correlations between the transfer factor of the lung for carbon monoxide (TLCO) and miR-126 and miR-145 were observed. A significant correlation has been found between serum miR-143 level and forced vital capacity (FVC). SSc patients with FVC ≤ 70% were characterized by significantly lower levels of miR-143 compared to patients with normal FVC. Additionally, the expression of miR-132 was significantly higher in dcSSc subgroup with detected active lung lesions compared to dcSSc patients with fibrotic lesions. Patients with an early scleroderma pattern of microangiopathy seen on nailfold video-capillaroscopy (NVC) revealed higher expression of miR-155 in serum than those with a late pattern. **Conclusions:** The expression profile of circulating cell-free miRNAs is significantly changed in the serum of SSc patients compared to healthy individuals. Downregulation of miRNA-181a and overexpression of miR-132, miR-143, miR-145 and miR-155 in serum may be significant in SSc in the context of biomarkers.

## 1. Introduction

Systemic sclerosis (SSc) is a chronic connective tissue disease of complex etiology. It is characterised by immune dysregulation, microvascular damage and fibrosis of the skin and internal organs. The clinical manifestations of SSc vary with different disease course and treatment response. According to the extent of skin thickening, the disease is classified as one of 2 subtypes: limited SSc (lcSSc), with skin involvement often restricted to sclerodactyly, and diffuse SSc (dcSSc), affecting areas of the skin proximal to elbows and knees [1].

Despite recent advances, making an early diagnosis of the disease and predicting its course still represent major clinical challenges for physicians. A delay in diagnosis results in a prolonged time to treatment initiation. The difficulties in predicting prognosis may lead to treatment inadequacy and irreversible organ dysfunctions. Therefore, there is an urgent need to develop new diagnostic methods in SSc.

However, much uncertainty still exists about the pathogenesis of SSc. It is not understood whether the process of pathogenesis is initiated by autoimmune system disbalance, disturbances in endothelial cells, or fibroblast dysfunction, as well as whether these three areas of dysfunction are associated and how the interactions between them lead to disease development [2]. The largest study of twins to date indicated that the occurrence of the SSc disease in both siblings was observed relatively rarely (4.7% of cases) in both monozygotic and dizygotic twins. Therefore, this study may suggest a stronger impact of environmental factors than genetic predisposition in the pathogenesis of SSc [3].

Fibrosis in SSc is caused by chronically activated myofibroblasts, and constant activation of fibroblasts is enhanced by interleukin 6 (IL-6), platelet-derived growth factor (PDGF), and transforming growth factor-beta (TGF-β), released by the immune and vascular systems [4]. As a result, excessive production of ECM manifests as fibrosis of the skin and internal organs. Generally, in the fibrosis process, three pathways play a crucial role: 1) TGF-β, 2) Wnt/β-catenin and 3) PPARγ. Both TGF-β and Wnt/β-catenin stimulate each other through the SMAD pathway, whereas PPARγ, by activating SMAD7, decreases the SMADS pathway and TGF-β signalling. On the other hand, TGF-β stimulates the WNT/β-catenin pathway and concurrently downregulates PPARγ expression. Therefore, PPARγ agonists can prevent fibrosis [5,6]. Another aspect of SSc, partially mentioned above, is vasculopathy and defective angiogenesis, which is a consequence of endothelial cell injury, among others [2]. At the earliest disease stages, increased levels of vascular endothelial growth factor (VEGF) are observed [7], and fibroproliferative events of the vessel occur [8]. High serum levels of circulating von Willebrand (VW) factor noted in the serum of SSc patients are also one of the hallmarks of endothelium injuries [9]. The third major and complement factor of SSc development is the immune system, which may be triggered by, e.g., apoptosis of endothelial cells, but also by environmental factors, such as cytomegalovirus [10] or Epstein–Barr Virus infection [11]. Interestingly, one of the hypotheses for the pathogenesis of autoimmune diseases leads to the Toll-like receptors (TLRs) theory. Normally TLRs recognize specific molecular patterns associated with different types of pathogens. However, inappropriate triggering of the TLR pathway by exogenous or endogenous ligands can lead to the initiation and/or perpetuation of autoimmune responses and tissue damage [12].

One of the posttranscriptional epigenetic mechanisms regulating the expression of the above-mentioned genes is microRNA. Moreover, free-circulating miRNAs in serum that may presumably derive from the degradation of numerous cells, such as endothelial cells and blood cells, during pathological processes may be valuable markers of disease and its clinical course. Regarding SSc, the available publications are mainly focused on tissue expression of miRNAs, with not enough data on the miRNA profiles in biofluids. Identifying blood-based biomarkers is essential due to their easy access and stability [13,14]. Therefore, the aim of this study was to determine the usability of selected circulating miRNAs as potential biomarkers in SSc patients and their association with the clinical course of disease and type of SSc. The common point in deciding which microRNAs to consider in this study was their involvement in the fibrosis process and their role in the TLR signaling pathway.

## 2. Results

### 2.1. SSc Patients Show a Defined Demographic and Clinical Profile

The disease duration varied from 6 months to 38 years (median 6 years) and was significantly longer in the limited subgroup (*p* = 0.008). Most of them were women in their 50s. About half of the patients were on immunosuppressive therapy. Relying on BMI, half of the patients presented excessive body mass, 22% were overweight, and 28% had obesity. The clinical presentation of SSc was variable (Table 1).

The clinical features present in all SSc patients were Raynaud’s phenomenon and skin fibrosis (Table 2). The most common manifestations included interstitial lung disease, present in 67% of patients, gastrointestinal tract involvement, detected in 49% of patients, and cardiac disease, confirmed in 38% of patients. The analysis of the degree of skin sclerosis showed that in most patients, the skin involvement was mild (mRSS 1−14). The extent of skin fibrosis was significantly more severe in patients with diffuse cutaneous systemic sclerosis (*p* = 0.01). In nearly 90% of patients, an abnormal capillaroscopic picture was demonstrated. The most common capillaroscopic patterns among the patients with lcSSc were early and active patterns. Among dcSSc patients, active and late patterns were the most frequently present. In more than half of patients, elevated ESR levels were found, while in 13% of patients, increased CRP was observed. Antinuclear antibodies were detected in 93% of patients. Antibodies against topoisomerase I were the most frequent (54% in SSc patients, 82.35% in dcSSc subtype and 33.3% in lcSSc). At the time of sample collection, more than half of the patients took immunosuppressive drugs (Table S1), mainly methotrexate (Table S2). A total of 80% of patients required vasodilatory therapy, of which 78% took amlodipine per os and 11% were receiving intravenous alprostadil.

### 2.2. Upregulation of miR-132, -143, -145 and -155 and Downregulation of miR-181a in the Serum of SSc Patients

**Figure 1:**
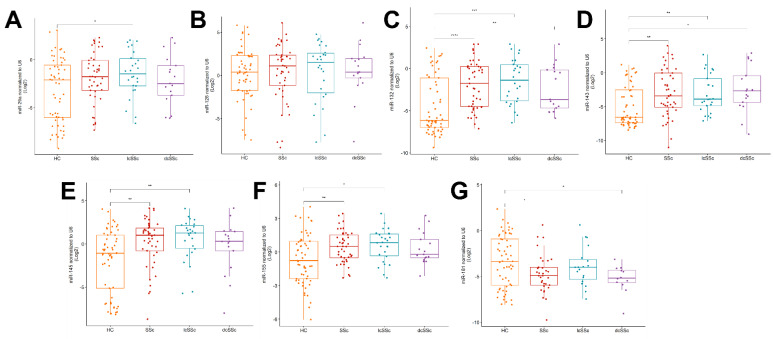
Expression of (**A**) miR–29a; (**B**) miR–126; (**C**) miR–132; (**D**) miR–143; (**E**) miR–145; (**F**) miR–155; (**G**) miR–181a in serum in healthy controls (HCs), systemic sclerosis (SSc) and the subtypes of SSc, limited systemic sclerosis (lcSSc) and diffusive systemic sclerosis (dcSSc). Data are presented with the median as the scattered boxplot graph. Pairwise posthoc significant differences: * *p* < 0.05; ** *p* < 0.01; *** *p* < 0.001; **** *p* < 0.0001.

Expression of all of the analyzed microRNA, excluding miR-29a and miR-126, was significantly different in SSc patients when compared to the healthy subjects. miR-3148 expression was observed neither in the study group nor in the controls. The relative expression of miR-126 was higher compared to the other analyzed microRNAs, both in SSc patients and in healthy subjects. However, the expression of serum miR-126 was characterized by high individual variability, and no statistically significant difference between patients and healthy subjects was noted (Figure 1B). miR-29a was significantly higher only in lcSSc patients compared to the healthy subjects (Figure 1A). Patients with SSc in comparison to HC were characterized by the upregulated level of miR-132, which was also seen when comparing both types of SSc separately to HC. However, there is no difference in miR-132 expression between lcSSc and dcSSc (Figure 1C). Additionally, miR-143, miR-145 and miR-155 were significantly higher in SSc and lcSSc but not dcSSc patients when compared to HC (Figure 1D–F). miR-181a was significantly downregulated in SSc patients when compared to the healthy group. Interestingly, this microRNA was detected only in one patient with dcSSc, whereas 42% of patients with lcSSc were characterized by its very low expression (Figure 1G).

**Figure 2:**
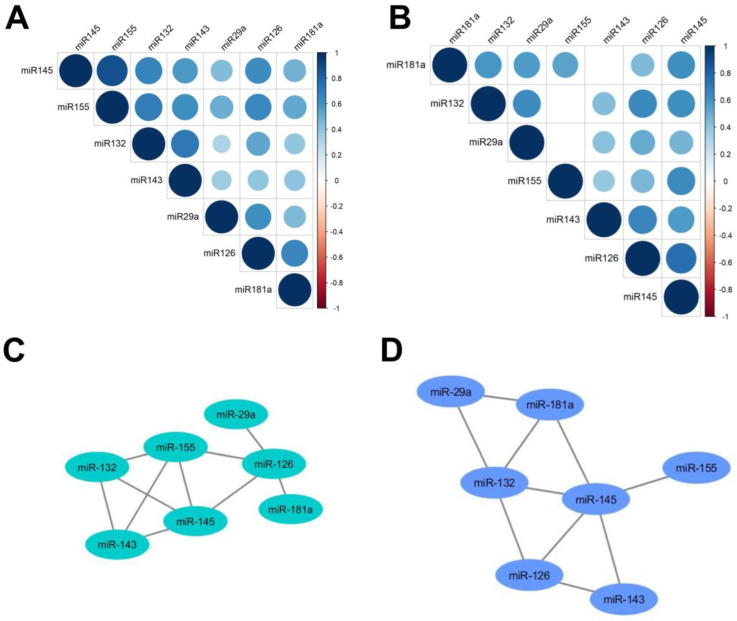
Correlation heatmap of analyzed microRNA levels in serum in (**A**) healthy subjects and (**B**) patients with systemic sclerosis. Only statistically significant correlations are shown with a circle. Below, network of high, statistically significant correlations (r ≥ 0.6) between analyzed microRNA levels in serum in (**C**) healthy subjects and (**D**) patients with systemic sclerosis. Spearman correlation test.

Additionally, in SSc patients as well as in healthy subjects, statistically significant high correlations (r ≥ 0.6) between serum levels of miR-145 and miR-126/miR-132/miR-143/miR-155 have been noted. In SSc patients, correlations between miR-29 and 181a/132, miR-181a and miR-132/miR-145 (Figure 2D) have been revealed, whereas in healthy subjects, correlations between miR-126 and miR-29a/181a/155 and miR-132 and miR-155 occurred (Figure 2C). Detailed values of correlation are presented in the Supplementary Material (Table S3).

### 2.3. microRNA and Clinical Association-Specific microRNAs Correlate with Microvascular Damage, TLCO and FVC

The present study did not reveal a correlation between the expression level of analyzed miRNAs and the age of the patients. No significant correlations were noted for inflammatory parameters such as ESR and CRP, as well as rheumatoid factor (RF).

**Figure 3:**
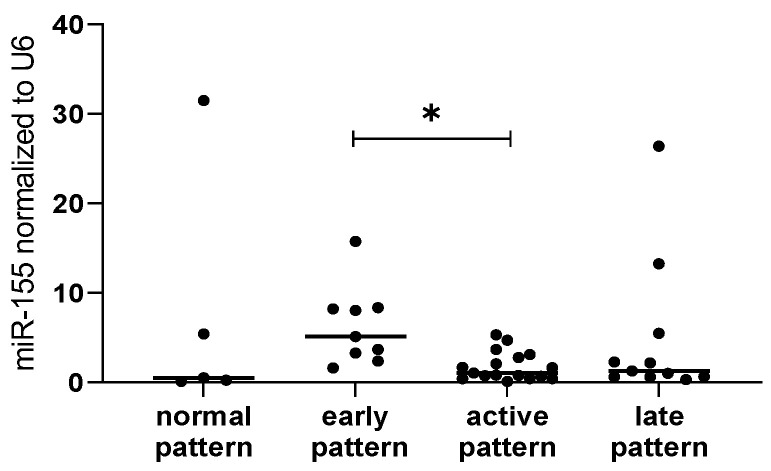
miR-155 expression level in systemic sclerosis patients with different capillaroscopy patterns. Data are presented with the median as scatterplot; * *p* < 0.05.

Based on capillaroscopy, it has been shown that the expression levels of miR-155 varied between patients with different degrees of microvascular damage among all SSc patients (*p* = 0.02). These differences were not observed when analyzing only patients with lcSSc or dcSSc (Figure 3).

Patients with dcSSc more frequently suffered from interstitial lung disease than lcSSc patients. The expression level of miR-132 differed between patients with different HRCT images in the dcSSc group (*p*-value = 0.02) but not in lcSSc or SSc patients. However, it has to be noted that the size of the subgroups was small (HRCT = 0, *n* = 3; HRCT = 2, *n* = 9 and HRCT = 5, *n* = 4), and in dcSSc patients with normal HRCT results and with fibrotic lesions (HRCT = 5), the expression of miR-132 was below quantification.

**Figure 4:**
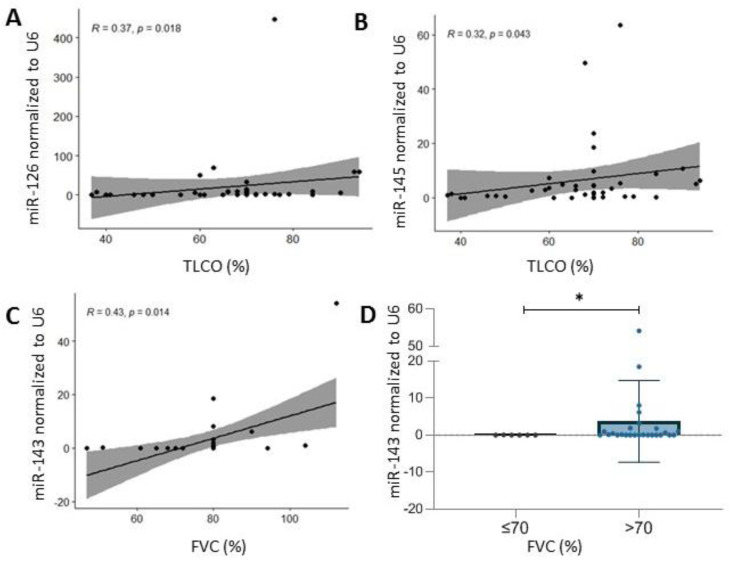
(**A**) Correlation between transfer factor for carbon monoxide (TLCO) and serum miR-126 level in systemic sclerosis patients. (**B**) Correlation between TLCO and serum miR-145 level in systemic sclerosis patients. (**C**) Correlation between forced vital capacity (FVC) and serum miR-143 level in systemic sclerosis patients. (**D**) miR-143 expression level in systemic sclerosis patients with forced vital capacity (FVC) equal to or below 70% and above 70%. Data are presented with the mean as a scatter dot plot; * *p* < 0.05.

The present study revealed correlations between the TLCO, associated with the ILD extension, and expression levels of miR-126 (r = 0.37, *p* = 0.02) (Figure 4A) and miR-145 (r = 0.32, *p* = 0.04) (Figure 4B) in SSc patients. Considering type of disease, these correlations were not observed. Additionally, the expression level of analyzed microRNAs in serum was compared between patients with TLCO < 70% and >70%, and significant differences were not noted.

The expression level of miR-143 correlated with the forced vital capacity (FVC) (r = 0.43, *p* = 0.014) (Figure 4C). Patients with >70% FVC were characterized by significantly higher levels of miR-143 (*p* = 0.03) (Figure 4D).

## 3. Discussion

SSc is an example of immune dysregulation and chronic inflammation whose pathogenesis is still not fully understood. SSc is characterized by fibrosis of the skin and internal organs and vasculopathy [1]. Because of the significant mortality associated with SSc, early diagnosis and treatment are crucial. However, the lack of validated markers of disease activity is still a clinical challenge. Similarly, the issue of objective assessment (e.g., by a molecular test) of the efficiency of therapy is not solved satisfactorily. Additionally, overlapping clinical presentations with phenotypic variations render the diagnostic process notably challenging. Therefore, much research is currently underway to find potential biomarkers of the disease, and one of the molecular targets is miRNAs. Most investigations focusing on miRNA as biomarkers consider serum or plasma as the most readily available and promising reservoir of miRNAs [15]. Our previous research has identified a number of miRNAs in serum as potential markers of a variety of ACTD, including MCTD, SLE and RA. The miR-132 expression profile in serum was one of the analyzed microRNAs potentially distinguishing SSc from other ACTD, such as RA or SLE [16]. Another study revealed that patients with SSc were characterized by upregulated miR-483-5p in serum, which seems to be associated with a disbalance of collagen type IV production [17]. Analysis of cell-free miRNAs in SSc plasma has shown downregulation of a combination of the miRNA-17~92 cluster in comparison to SLE patients [18].

The common denominator for the selection of the analyzed microRNAs in the present study was their participation in the fibrosis process and their role in the TLR signaling pathway. The present study revealed upregulated profile of most of the selected serum microRNAs (excluding miR-29a, miR-126 and miR-181a) in comparison to healthy subjects. An increased expression of most of the analyzed microRNAs studied in lung tissue of SSc patients was also shown by Huang et al. [19]. Therefore, the observed disbalance of the microRNAs expression pattern must be certainly related to the pathological background of the disease. Moreover, the present paper also revealed different pair-wise correlations between miRNA levels in healthy subjects and SSc patients. Interestingly, the authors of a recent meta-analysis of differentially expressed microRNAs in SSc noted that most of the researchers focused on downregulated miRNAs rather than upregulated ones. Besides, most studies were conducted on samples of dermal fibroblast but not blood samples [20]. Contrary to our observation of upregulated serum level of miR-29a in lcSSc patients, research on SSc-cultured fibroblasts, SSc skin biopsies and mouse models revealed a decrease in miR-29a expression [21]. Nevertheless, Wuttge et al. noticed that plasma miR-29a levels in SSc patients depend on specific autoantibody profiles. The authors revealed that plasma miR-29 expression is downregulated in patients with anti-centromere antibodies (ACA) in comparison to the plasma level observed in patients with U1-RNP antibodies [22]. However, in the present study, we did not reveal an association between analyzed miRNAs level and antibodies. Most of the analyzed patients were anti-TOPO-positive, particularly patients with dcSSc, which is a characteristic feature of this subtype of the disease [23].

In the present study, we also compared microRNA expression patterns that could discriminate between diffusive and limited SSc. It is crucial to note that miR-181a was detected only in the serum of patients with lcSSc. The involvement of miR-181a in the process of fibrosis and modulation of inflammatory response via TLR4 or TGF-β pathways [24,25] reveals the importance of this microRNA in the development of SSc. Similarly, Chouri et al. described a higher level of miR-181a in lcSSc than in dcSSc [17]. At this point, it is important to note the limitation of the present study. Due to the fact that SSc is a rare disease, we could not conduct this analysis on a larger group of patients. Another more important constraint is that the patients were characterized by different disease activity, damage index and treatment. Nevertheless, in our further studies, we have to discover whether the miR-181a release into serum was associated with the type of SSc or disease activity or treatment. Contrary to our outcomes, the analysis of miR-181a in systemic lupus erythematosus revealed its increased expression in plasma in comparison to healthy subjects [26,27]. Observed discrepancies may stem not only from the different disease entities but also from sample types, which have to be taken into consideration when planning the application of microRNAs as a potential diagnostic test.

This study was also undertaken to find associations between microRNA level in serum with clinical parameters of SSc.

Since the typical capillaroscopic image reflecting microvascular damage is highly specific for SSc, it has been included as one of the elements evaluated in the current ACR/EULAR SSc Classification Criteria [28]. Based on the Cutolo classification, three major NVC patterns are observed: (1) early (well-preserved capillary distribution but with a few enlarged/giant capillaries and capillary haemorrhages), (2) active (moderate capillary loss, mild disorganization of capillary architecture, frequent giant capillaries and capillary haemorrhages), and (3) late (significant capillary loss with large avascular areas, disorganization of the normal capillary architecture, branching/bushy capillaries, irregular capillary dilation, and few or absent giant capillaries and hemorrhages) [29]. In the present study, we noticed a significantly higher level of serum miR-155 in patients with early patterns of microvascular damage when compared to the SSc patients with active patterns. Overexpression of miR-155 leads to pathological processes [30], which is consistent with our findings where SSc patients were characterized by higher levels of miR-155 than healthy subjects. Furthermore, it has been described as a pro-fibrotic factor in skin and lungs in SSc patients [31,32,33], but also, the role of miR-155 on resistance and permeability of endothelial cells has been proven [33], along with its proarteriogenic function [34]. Moreover, its role in proper B and T lymphocyte and dendritic cell function and antibody production has been well known for many years [35,36]. Therefore, the interplay between vascular endothelial cells and immune cells is important in terms of pathogenesis and disease progression.

Another severe aspect of SSc is lung involvement, which is one of the most common causes of mortality among patients with this disease. Unfortunately, functional tests and imaging examinations of initial lung lesion severity do not provide sufficient data to accurately predict the further progression of interstitial lung disease in SSc (ILD-SSc). Therefore, the dynamics of forced vital capacity (FVC) and transfer factor of the lung for carbon monoxide (TLCO) over time play an important role as a predictive factor [37]. The presence of finger ulcers and pulmonary hypertension are considered clinical parameters that are poor prognostic factors associated with a more rapid decline in TLCO [38]. Hence, there is an emerging need to identify other prognostic markers to predict if severe pulmonary involvement occurs. The present association study revealed a positive correlation between TLCO and the serum level of miR-126 and miR-145. Generally, it is assumed that miR-126 is one of the antifibrotic factors [39] and plays a role in angiogenesis [40]. miR-126 seems to negatively regulate the expression of the Epidermal Growth Factor Like-domain 7 gene (EGFL7) in fibroblasts. Moreover, according to Liakouli et al., due to EGFL7’s implications for collagen production, its impaired expression is probably one of the pathogenesis factors for SSc [41]. Interestingly, in the case of miR-126 expression, we did not find a statistically significant difference between SSc patients and healthy subjects; however, it has to be mentioned that intra-individual variability of miR-126 expression was quite broad.

The second microRNA associated with the TLCO parameter in the present study was miR-145. Similarly to miR-126, miR-145 is also considered an antifibrotic factor [42]. Moreover, Zhu et al. identified miR-145 as one of those microRNAs that is significant in SSc fibrosis, and its level was decreased in skin tissues and fibroblasts. The authors confirmed its role via SMAD3 downregulation, thereby affecting the TGF-β signalling pathway [42]. Recently, another novel pro-fibrotic miR-145/KLF4 pathway was identified by Ly et al. [43]. KLF4 is a transcription factor Kruppel-like factor 4, of which decreased expression was noted, particularly in the early stages of SSc [44] but also in human fibrotic liver [45]. Moreover, the reduction of KLF4 expression can be partially caused by TGFβ and is probably the point of WNT-induced pro-fibrotic programmes [46]. Mir-145 and miR-143 form a cluster that, by definition, means they are transcribed in the same orientation [47]. However, a lack of homology in their mature sequence indicates their ability to bind to and regulate different targets [48]. Nevertheless, the present study revealed a significant correlation between serum miR-143 level and forced vital capacity (FVC). Moreover, SSc patients with abnormal FVC results were characterized by significantly lower levels of miR-143 compared to patients with normal FVC. In the present study, healthy subjects were characterized by lower levels of miR-143 than SSc and lcSSc but not dcSSc patients. This result seems to be contrary to the outcomes described by Christmann et al. [32]. However, the differences that occurred are probably a result of the difference in the used type of sample. Christmann used PBMC and lung tissue. Recent studies may indicate contradictory effects of miR-143. For example, Tu et al. revealed that microRNA-143-3p attenuated the development of hepatic fibrosis in autoimmune hepatitis through regulation of TAK1 phosphorylation [49], whereas Diazzi et al. described cluster miR-143/miR-145 as pro-fibrotic [50]. Another aspect of the potential role of miR-143 in SSc pathogenesis is its role in the TLR signalling pathway [51] and regulation of IL-6 and IL-8 secretion [52].

Our study also confirmed the significant upregulation of miR-132 in the serum of dcSSc patients with active lung lesions compared to patients with fibrotic changes. Considering the fact that the ILD may potentially respond to treatment only at the stage of active lesions, it is crucial to select the patients before irreversible fibrosis appears. Although these results are very encouraging, it should be noted that due to the small number of patients in each subgroup, they should be considered exploratory data. Growing literature reveal its importance in renal [53], cardiac [54,55] and liver fibrosis [56]. Interestingly, in one of the studies, the expression of miR-132 in fibroblasts from the SSc patients’ skin revealed a lower level of this miRNA than in healthy subjects [57]. In livers with alcohol fibrosis or cirrhosis, mir-132 reveals significantly higher expression compared to the healthy group [56]. Our study also revealed upregulated serum levels of miR-132 in SSc patients in comparison to the healthy subjects.

The present study did not reveal the correlation between the type of therapy and the miRNA patterns in SSc patients. However, a growing body of literature has examined the potential influence of medications on the expression of microRNA with intriguing results. Amlodipine, a member of the dihydropyridine class of calcium channel blockers, broadly used in hypertension management, is the first-line drug recommended for the treatment of Raynaud’s phenomenon in SSc patients. Amlodipine has been shown to induce miR-21 overexpression in plasma [58] and to decrease miR-155 expression in human umbilical vein endothelial cells (HUVECs) [59]. There are several potential reasons why our study has not confirmed the previous results. The prime cause of the discrepancy is due to the fact that the above-mentioned studies were conducted in China, where S-amlodipine, an S-enantiomer of amlodipine, is available [60]. The impact of the amlodipine conventionally used in Europe, which is a mix of S- and R-enantiomers on serum microRNA expression, is still to be verified [61]. Several authors have also highlighted the potential influence of immunosuppressive drugs on miRNA levels. Iwamoto et al. revealed that miR-877-3p was upregulated in response to methotrexate in rheumatoid arthritis fibroblast-like synovial cells [62]. Experiments conducted by Yang et al. also suggest that methotrexate and its downstream metabolite adenosine significantly upregulates miR181b expression in cultured human umbilical vein endothelial cells [63].

Based on the properties of microRNAs, the potential for diagnostic purposes and also future therapeutics seems to be promising. However, the results of the study need to be interpreted with caution as depending on the organ or tissue, the effect of miRNAs on the fibrotic process still does not appear to be clear, and the findings are not consistent.

Moreover, as mentioned above, the present study was limited in several ways. Results of the studies on free circulating microRNAs in biofluids may vary due to the methods used. A major source of uncertainty is the lack of standardization methods from the isolation to further stages. Other aspects are a broad range of reference genes and sometimes inappropriate normalization to an exogenous spike-in; therefore, implementation of this type of research as a diagnostic tool is challenging [64,65]. Further data collection to assess larger groups of patients representing two subtypes of SSc, with a similar scheme of treatment and similar disease activity, would be highly recommended. Additionally, factors such as cytomegalovirus or Epstein–Barr Virus infection and different treatment regimens in SSc patients should be considered in further studies on microRNA expression. Nevertheless, we believe that our study has gone some way towards enhancing our understanding of the expression patterns in serum of SSc patients, despite the fact that the picture remains still incomplete.

## 4. Materials and Methods

### 4.1. Patients

The study was performed on SSc patients from the Department of Connective Tissue Diseases, National Institute of Geriatrics, Rheumatology and Rehabilitation, Warsaw, between 2016 and 2017. All SSc patients met the American College of Rheumatology/European Alliance of Associations for Rheumatology (ACR/EULAR 2013) classification criteria for SSc [28]. All participants provided their written informed consent approved by the local ethics committee. The study group consisted of 45 SSc patients (34 female and 11 male) and 57 healthy matched subjects (49 female and 8 male) aged 51 ± 13 and 50 ± 6.5 years old. All of the patients were of Caucasian ethnicity. The patients included in the study were classified as having limited SSc (*n* = 27; 60%) or diffuse SSc (*n* = 18; 40%), according to subtype criteria [1]. The exclusion criteria were defined as the presence of cancer, infection or pregnancy at the time-point of sample collection.

The patient characteristics were obtained at the time of the blood sample collection and included the following: disease duration (measured as the time from Raynaud’s phenomenon onset), body mass index, presence of Raynaud’s phenomenon, digital ulcers, musculoskeletal system, cardiovascular system, digestive system and renal involvement. In order to measure the severity of skin involvement, patients underwent examination using a modified Rodnan skin score. Using nailfold video-capillaroscopy (Dino-Lite Capillaryscope 200; Dino-Capture 2.0), the microvascular damage pattern was determined [66]. Screening for pulmonary involvement was conducted by a physical examination and chest x-ray imaging (Carestream Health DRX system, Radiology Department of National Institute of Geriatrics, Rheumatology and Rehabilitation, Warsaw, Poland). With the existing indications of SSc-ILD, high-resolution computed tomography (HRCT) was also performed using an Optima CT660 system (Mokotowskie Centrum Medyczne, Warsaw, Poland) and a Toshiba Aqualion 16 (Luxmed Diagnostyka, Warsaw, Poland). HRCTs were reviewed blindly from clinical data. ILD was classified as active in the presence of ground glass opacity and reticular opacities, while honeycombing was regarded as irreversible fibrotic lesions [67]. The study did not include a quantitative assessment of the lesions. Pulmonary function tests included FVC (forced vital capacity) and TLCO (transfer factor of the lung for carbon monoxide) and were conducted in the Department of Physiopathology of Breathing; Tuberculosis and Lung Diseases Institute, Warsaw, using MasterScreen Body/Diff Jaeger. The percentage of the predicted value for TLCO (TLCO%) was corrected for measured haemoglobin.

Laboratory blood tests, including erythrocyte sedimentation rate, C-reactive protein test and antinuclear antibody detection, were conducted in NIGRiR (Sedi System, Vitros 4600 (Ortho Clinical Diagnostics, Raritan, NJ, USA)).

### 4.2. Analysis of miRNA Expression

Total RNA, including miRNA, was isolated from 500 μL of serum using a miRNA Concentrator kit (A&A Biotechnology, Gdansk, Poland) and Tri-Reagent (Invitrogen, Waltham, MA, USA) according to the manufacturer’s instructions. In this study, to standardize samples, the same volume of serumw as taken. A TaqMan MicroRNA Reverse Transcription Kit (Applied Biosystems, Waltham, MA, USA) was used to conduct reverse transcription of miRNA. Additionally, preamplification was conducted using the TaqMan PreAmp Master Mix Kit (Applied Biosystems, USA). After that, the TaqMan Universal Master Mix II, UNG (Applied Biosystems, USA) and TaqMan Assays (Applied Biosystems, USA): miR-29a (assay ID 002112-3p), miR-126 (assay ID 002228-3p), miR-132 (assay ID 000457-3p), miR-143 (assay ID 002249-3p), mir-145 (assay ID 002278-5p), mir-155 (assay ID 002623-5p), mir-181a (assay ID 000480-5p) and hsa-miR-3148 (assay ID 465272_mat) were used for RT-PCR reaction. The reaction was performed on a QuantStudio 5 Real-Time PCR System (Applied Biosystems, USA). Each sample was analyzed in duplicates. U6 snRNA (assay ID 001973) was taken as the housekeeping gene. Relative expression of analyzed microRNAs was calculated by the ΔCt method and normalized using Log2(ΔCt) transformation.

### 4.3. Statystical Analysis

Two independent groups were compared using the nonparametric Mann–Whitney U test. Multiple comparisons of independent groups were conducted by Kruskal–Wallis test with Dunn’s posthoc. Correlations between microRNA-microRNA and microRNA expression and clinical parameters were checked by the Spearman test. The correlation matrix was computed and visualized using the R program and corrplot package: Visualization of a Correlation Matrix. GraphPad Prism 9.2.0 (GraphPad Software, San Diego, CA, USA) and R program (R Development Core Team (2008) R: A language and environment for statistical computing. R Foundation for Statistical Computing, Vienna, Austria ISBN 3-900051-07-0, URL http://www.R-project.org) were used for data analysis and figure preparation. Graphs were created using GraphPad Prism Software 9.4.0, and visualization of correlations network between miRNAs by Cytoscape 3.7.0 (https://cytoscape.org, Version 3.7.0).

## 5. Conclusions

The expression profile of circulating cell-free miRNAs is significantly changed in the serum of SSc patients compared to healthy individuals. Moreover, depending on the analyzed group (SSc patients or healthy subjects), different correlations between microRNAs were noted. Nevertheless, a study on the functional mechanism of the association between selected microRNAs is necessary. Downregulation of miRNA-181a and overexpression of miR-132, miR-143, miR-145 and miR-155 in serum may be significant in SSc in the context of biomarkers.

## Figures and Tables

**Table 1:** Demographic data and SSc patients’ characteristics.

Variable	SSc	lcSSc	dcSSc
Sex, female: *n* (%)	34 (75.56)	22 (48.89)	12 (26.67)
Sex, male: *n* (%)	11 (24.44)	5 (11.11)	6 (13.33)
Age, years: mean ± SD	51 ± 13	54 ± 10,4	47 ± 15
BMI	24.9 (17.1–40.9)	26.7 ± 5.9	24.2 (17.1–40.9)
Disease duration, years: median (min–max)	6 (0–38)	11 (1–38)	3 (0–20)
Immunosuppressive therapy: *n* (%)	28 (56%)	15 (51.85%)	13 (58.82%)
mRSS median (min-max)	7 (2–30)	5 (2–19)	11 (2–30)
Scleroderma pattern in NVC	37 (88.1%)	21 (84%)	16 (94.12%)
Presence of RF, *n* (%)	3 (9)	3 (15)	0
Hepatitis type B	0	0	0
Hepatitis type C	0	0	0
Elevated ESR, *n* (%)	24 (55)	14 (53.85)	10 (58.82)
ESR, median (min–max)	14 (2–78)	13 (2–78)	14 (4–53)
Elevated CRP, *n* (%)	6 (13)	4 (14.81)	2 (11.76)
CRP, median (min–max)	3 (1–52)	3 (1–52)	5 (1–45)
Presence of ANA, *n* (%)	43 (93.33)	25 (92.59)	18 (100)
ANA range (min–max)	1:80–1:40960	1:80–1:40960	1:320–1:10280
Anti-TOPO-I	21 (54%)	6 (33,3%)	15 (82,35%)
CENP	12 (31%)	12 (33.3%)	0 (0%)

CRP > 10 mg/L considered elevated; ESR > 12 mm/h considered elevated; ANA ≥ 1:160 considered aspositive; ANA: antinuclear antibodies; anti-TOPO-I: anti-topoisomerase I antibodies; BMI: body mass index; CENP: antibodies to centromere protein; CRP: C-reactive protein; ESR: erythrocyte sedimentation rate; mRSS: modified Rodnan skin score; NVC: nailfold video-capillaroscopy; RF: rheumatoid factor.

**Table 2:** Clinical manifestation in SSc patients.

Variable	lcSSc *n* (%)	dcSSc *n* (%)	*p* *	SSc *n* (%)
Raynaud’s phenomenon	27 (100)	18 (100)	>0.99	45 (100)
Digital ulcers	11 (40.74)	8 (44.44)	0.76	19 (42.22)
Digestive system involvement	12 (44.44)	10 (55.56)	0.77	22 (48.89)
Interstitial lung disease	15 (55.56)	15 (83.33)	0.10	30 (66.67)
Pulmonary arterial hypertension	2 (7.41)	1 (5.56)	0.36	3 (6.67)
Heart involvement	9 (33.33)	8 (44.44)	>0.99	17 (37.78)
Arthritis	10 (37.04)	6 (33.33)	0.73	16 (35.56)
Myositis	2 (7.41)	1 (5.56)	0.55	3 (6.67)
Renal involvement	3 (11.11)	1 (5.56)	>0.99	4 (8.89)

* Fisher’s test; comparison between lcSSc and dcSSc.

## Data Availability

The data presented in this study are available on request from the corresponding author.

## Supplementary Materials

The following supporting information can be downloaded at: www.mdpi.com/article/10.3390/ijms231810731/s1.
