# Supplementary material for: Serum microRNAs in Systemic Sclerosis, Associations with Digital Vasculopathy and Lung Involvement
Source: Int J Mol Sci. 2022 Sep 14;23(18):10731. doi: 10.3390/ijms231810731 (PMC9503032; doi:10.3390/ijms231810731)
Supplement: Supplementary file 1 [file ijms-23-10731-s001.zip › ijms-1874540-supplementary.pdf]

Table S1. Immunosuppressive agents used in the study group at the time of the blod sample collection.

Medications	lcSSc, n (%)	dcSSc, n (%)	SSc, n (%)
Methotrexate	7 (25,93)	4 (23,53)	11 (24)
Mycophenolate mofetil	2 (7,41)	5 (27,78)	7 (16)
Azathioprine	2 (7,41)	2 (11,76)	4 (9)
Glucocorticosteroids	4 (14,81)	2 (11,76)	6 (13)

Table S2. Different methotrexate dosages in a study group at the time of sample collection.

Methotrexate Dose (mg/week)	Number of Patients- n (%)
10	1 (9,09)
12,5	1 (9,09)
15	6 (54,55)
20	3 (27,27)

Table S3. Correlation between microRNA expression in healthy subjects and patients with SSc.

**Healthy Subjects**					**SSc Patients**				
microRNA interaction		*r*	*p*	*n*	*microRNA interaction*		*r*	*p*	*n*
miR145	miR155	0.879	0.000	57	miR126	miR145	0.755	0.000	45
miR132	miR143	0.712	0.000	57	miR126	miR143	0.655	0.000	45
miR132	miR155	0.697	0.000	57	miR126	miR132	0.647	0.000	45
miR132	miR145	0.664	0.000	57	miR145	miR155	0.637	0.000	45
miR126	miR181a	0.658	0.000	57	miR132	miR29a	0.635	0.000	45
miR126	miR155	0.641	0.000	57	miR132	miR145	0.615	0.000	45
miR126	miR145	0.625	0.000	57	miR145	miR181a	0.614	0.000	33
miR126	miR29a	0.619	0.000	57	miR132	miR181a	0.589	0.000	33
miR143	miR155	0.618	0.000	57	miR143	miR145	0.570	0.000	45
miR143	miR145	0.575	0.000	57	miR181a	miR29a	0.566	0.001	33
miR126	miR132	0.520	0.000	57	miR155	miR181a	0.534	0.001	33
miR155	miR181a	0.511	0.000	57	miR126	miR29a	0.506	0.000	45
miR155	miR29a	0.497	0.000	57	miR145	miR29a	0.464	0.001	45
miR145	miR181a	0.471	0.000	57	miR126	miR155	0.456	0.002	45
miR181a	miR29a	0.447	0.000	57	miR126	miR181a	0.443	0.010	33
miR145	miR29a	0.438	0.001	57	miR132	miR143	0.433	0.003	45
miR143	miR181a	0.405	0.002	57	miR143	miR29a	0.411	0.005	45
miR126	miR143	0.399	0.002	57	miR143	miR155	0.383	0.009	45
miR132	miR181a	0.397	0.002	57	miR143	miR181a	0.342	0.051	33
miR143	miR29a	0.356	0.007	57	miR155	miR29a	0.281	0.062	45
miR132	miR29a	0.301	0.023	57	miR132	miR155	0.263	0.081	45

* Spearman correlation
